# Public health messages during a global emergency through an online community: a discourse and sentiment analysis

**DOI:** 10.3389/fdgth.2023.1130784

**Published:** 2023-06-28

**Authors:** Megan Watkins, Jaimee S. Mallion, Daniel Frings, Jane Wills, Susie Sykes, Andrew Whittaker

**Affiliations:** ^1^ Institute of Health and Social Care, London South Bank University, London, United Kingdom; ^2^ School of Applied Sciences, London South Bank University, London, United Kingdom

**Keywords:** Facebook (FB), online community, discourse, sentiment, longitudinal, COVID-19

## Abstract

The growing popularity of social media and its ubiquitous presence in our lives brings associated risks such as the spread of mis- and disinformation, particularly when these may be unregulated in times of global crises. Online communities are able to provide support by enabling connection with others and also provide great potential for dynamic interaction and timely dissemination of information compared with more traditional methods. This study evaluates interactions within the Essex Coronavirus Action/Support Facebook private group, which set out to prevent the spread of COVID-19 infection by informing Essex residents of guidance and helping vulnerable individuals. At the outset, 18 community administrators oversaw the group, which attracted approximately 37,900 members. Longitudinal Facebook group interactions across five periods spanning the UK lockdowns 2020–2021 were analysed using psychological discourse analysis and supplementary computed-mediated analysis to further explore sentiment and linguistic features. The findings endorsed that the group provided a protected space for residents to express their feelings in times of crises and an opportunity to address confusion and concern. The effective communication of public health messages was facilitated by promoting desired interaction and the construction of group identities. Administrators worked with group members to achieve a shared understanding of others' perspectives and the COVID-19 evidence base, which led to a mobilisation of the provision of support in the community. This was accomplished through the application of rhetorical and interactional devices. This study demonstrates how online groups can employ discursive strategies to engage audiences, build cohesion, provide support, and encourage health protective behaviours. This has implications for public health teams in terms of designing, implementing, or evaluating such interventions.

## Introduction

In the context of a crisis, people tend to draw on personal information and communication technology for creative responses and problem-solving. Trusted and credible informational channels are pursued as a means of coping with uncertainty (1). Social media can be amenable to this given its popularity, ability to regulate, and address misinformation quickly. Unregulated social media, however, poses health risks, particularly in the context of a pandemic (2). Studies have found that COVID-19 conspiracy beliefs and use of social media as a source of information were positively related, whilst COVID-19 conspiracy beliefs and health-protective behaviours were negatively related (3). Within the context of public communication during global crises, there is a need for precise and effective public health communication. In line with this, social media platforms and the UK government introduced a package of measures with the intention of reducing vaccine disinformation (4), which encouraged platforms to work with public health bodies to promote the dissemination of accurate messages.

Some social media application models, which employed peer support, were presented for disaster outreach during the COVID-19 outbreak (5). In terms of platforms used, there is evidence to suggest that Instagram, more so than Twitter, has been shown to be useful in promoting meaningful, interactive communication during global health crises (6). This was especially the case when based on communication principles like solution-based messaging and acknowledging fears/concerns. Whilst this work focused on the Ebola outbreak, it mirrored later strategies proposed to improve public message development in response to COVID-19 (7). This work advocated that communication at the individual, health system, and population level should include engaging the audience as partners, communicating with compassion transparency, honesty, and frequent evaluation. For example, a sentiment analysis of tweets during the COVID-19 crisis reported that positive and trust emotions were most commonly found in communications about, or the issuing of, guidance (8).

The value of computer-mediated communication and online groups in providing social support has been recognised. Online social support has been reported to be significantly related to both online social ties and altruism (9) and occurs through the facilitation of support-seeking processes. Support seeking is enabled by the creation of a comfortable context and safe space to elicit help, in which there is greater access to those capable of, and willing to, provide support (10). Little is known, however, about how social media platforms have been utilised by public health organisations in infectious disease outbreak scenarios (11).

Social media has brought about societal change in the speed and nature of communication (12). Dynamic methods for communicating public health messages (13) may assist in overcoming barriers to dissemination. Whilst social media platforms have been employed in recent years for the delivery of health promotion, the aims of public health interventions using social media have differed greatly (14). Researchers and practitioners must better understand how to utilise social media effectively (15) in terms of strategies that can be used to engage audiences and encourage best practices. The effects of specific features can be usefully investigated in order to guide behaviour change intervention design (16). The literature identifies a need for better quality social media interventions with adequate intervention descriptions and use of contemporary platforms (17, 18).

Social media users may have a range of responses to content they engage with on contemporary platforms and these responses would influence their receptivity to any messaging using these platforms. An impactful component of social media groups, differing from traditional broadcast channels, may be the possibility for members to visibly express negative emotional reactions to information received. Negative online comments have been found to decrease readers' perceived credibility of the corresponding news. This may be attributable to negative comments holding perceptual superiority and being more memorable than positive comments (19). Exposure to negative comments is, however, more likely to influence individuals who perceive the comments to be authentic.

Anger, as a more specific negative emotional state, can range from mild irritation to rage upon encountering reactance-inducing messages that violate individual choice or autonomy (20). Expressions of anger online may indicate daily affect in people's lives. A linguistic inquiry and word count study investigating open-ended diary responses found that anger expressions were more related to daily experienced anger than expressions of anxiety, sadness, depression, and positive emotion and their corresponding daily affect (21). In some cases, anger has been described as a strong driver of political participation and appeals to anger have been found to strongly predict engagement with Facebook posts (22).

Empathy, which has been recognised as both a cognitive and a communication resource (23), may be elicited and received following the expression of negative emotions and the hypothesising of the mental state. Interactional episodes providing an opportunity to express empathy and support can promote the development of relationships and collegiate communications online. For example, team members discussing technological problems shared experiences with, and gave advice to, one another (24). In accordance, language associated with the threat to reputation may have the converse effect on interpersonal relationships. For this reason, we may save our face and that of others to achieve harmonious discourse (25).

A lack of trust in authorities can perpetuate social agency when individuals, by acting together, form a joint identity (26). Dominant patterns of talk associated with mobilisation on Facebook event pages have included the disputing of the integrity of authorities, often contrasting with the creation of a positive atmosphere and “togetherness” (27). Particular devices, such as pronouns, have been implicated in the construction of different combinations of identity and alignment with different standpoints. A discourse analysis showed that patterns of agreement within an online graduate course were related to shared understanding and cohesion, and community development was promoted through the use of inclusive language (28).

Ambiguous identity may impede relationship building within online groups. It has been recommended that social media users, to benefit individuals and organisations, should avoid identity obfuscation within posts (29). In accordance, authenticity can be an effective discursive strategy within many contexts; it can promote relationship development and effective communication about health contributing to change in therapeutic settings (30), and within the workplace, authenticity can frame talk as unscripted with genuine intentions (31).

The present study investigates the specific discourse and linguistic features of a particular online community intervention during the context of a pandemic. In March 2020, the Essex Coronavirus Action/Support (ECAS) Facebook page was set up (32) for disseminating information to the community, gaining around 55,000 followers. A private group was also created, initially attracting around 37,900 members (33). It was described as a space for Essex residents and encouraged connection and discussion around the COVID-19 pandemic, focusing on preventing the spread of infection of COVID-19, informing Essex residents of guidance, and assisting vulnerable individuals. ECAS collaborates with the Essex Public Health Team at Essex County Council (ECC) and local Facebook groups. The collaboration was initiated by ECC. ECAS has since branched out to address further issues and is now known as Essex is United.

This paper addresses the following questions: What were the features of group discourse during the COVID-19 pandemic that developed and maintained connectedness? Which discursive strategies facilitated effective communication of public health guidance? What encouraged group members to engage in desired interaction and mutual aid? These areas of enquiry form part of a wider evaluation of the ECAS group to establish how practice could contribute towards achieving whole system change for the public health function.^1^

This study contributes to the evidence base regarding specific features and actionable strategies for the dissemination of public health messages through an online community, specifically during a global crisis.

## Methods

### Ethical approval

Ethical approval for this study was granted by the School of Health and Social Care Ethics Committee at London South Bank University (LSBU; references ETH2021-0148 and ETH2021-0149). University guidelines were followed.

The key ethical consideration for this study was the detailed analysis of Facebook group postings without participants' explicit consent. The LSBU code of practice for human research 2020, sections 2.1 and 2.2 (34), recognises the balance between the need for consent in such studies but also the principle of fair processing and that a judgement regarding the potential for use of data to cause distress is needed.

As recommended by Stommel and Rijk (35), following their investigation of the reporting of ethical issues surrounding online data by discourse analysts, a Public Involvement and Engagement (PIE) panel, comprised five ECAS group members, was involved in ethical decision-making. Feedback suggested that analyses without explicit consent would be reasonable if the data were anonymised in publications, and it was highlighted that, although the Facebook group is technically a closed group, it is a “semi-public” space given the large number of members. Furthermore, the only reason that the group is closed to an individual is premised upon the geographical catchment area. Permission from the site owners for analyses was obtained.

Nevertheless, consideration should still be given to the potential retrievability of posts despite the anonymisation of quotes in this paper. Approved group members with access to group content may be able to access these, and thus, example quotations to build a case have been selected sensitively.

### Participants

When membership of the ECAS group is requested, administrators ask for a postal code to verify that applicants are Essex residents and members are not actively recruited. Whilst approximately 80 per cent of the group members are women, men have been reported to be over-represented in discussion within comments (36). At the outset, 18 community administrators oversaw the private group (with approximately 37,900 members) and three had a prominent role, with ECC paying for their work.

### Data collection

**Figure 1:**
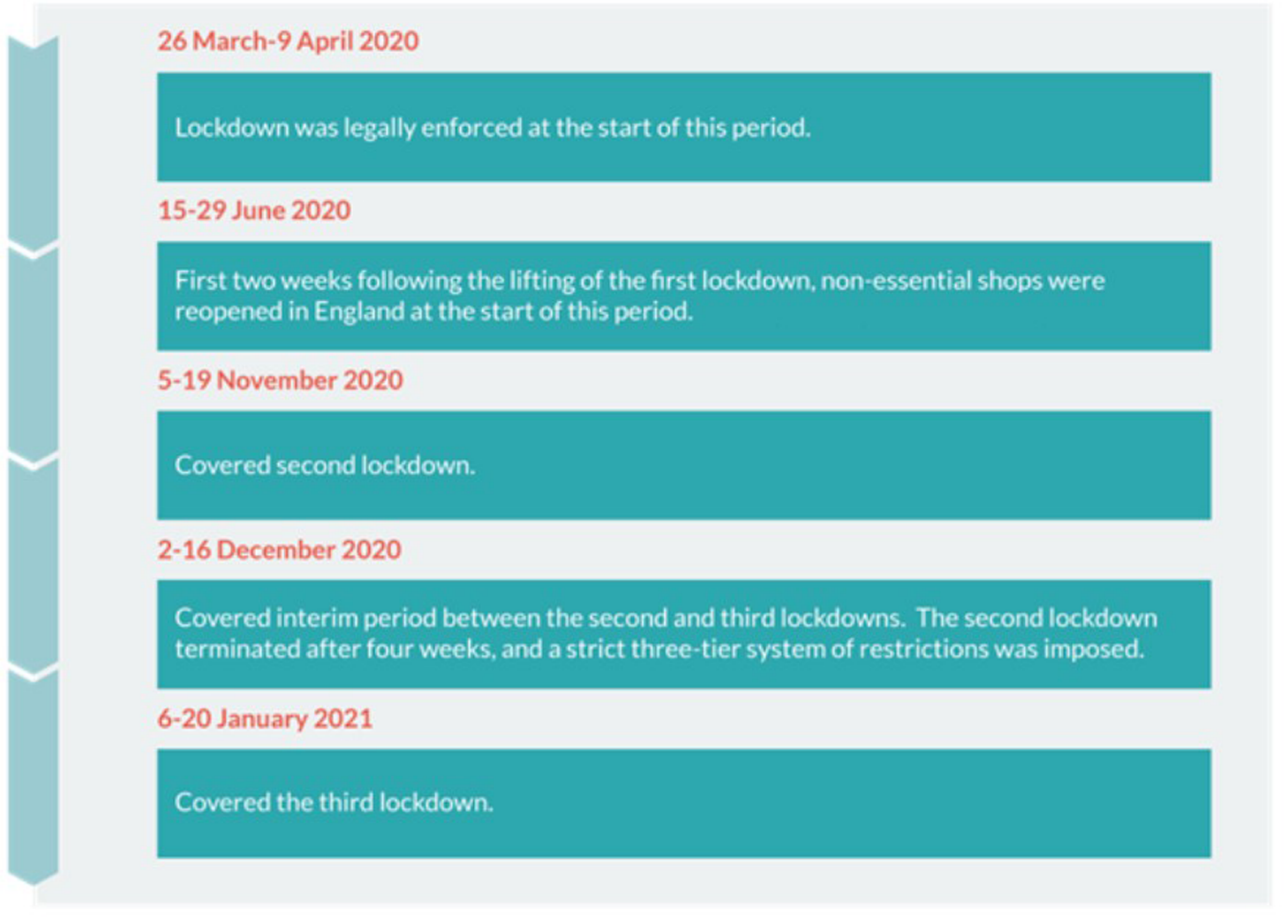
Time periods for investigation.

Five salient time periods were identified within coproduction workshops with the ECAS team and selection was also informed by the timeline of UK lockdowns (37). Each time point covered a 2-week period across the first, second, and third lockdowns and two interim periods of eased restrictions (see Figure 1 for specific dates). Selection of these specific periods allowed the development of the group to be examined over time to address the research questions. The ECAS Facebook Group was searched, using the Facebook search function, for relevant months, and interactions for the specified periods were manually harvested (copied into a Microsoft Word document). The files were then imported to qualitative software and formed the corpus for analysis. The authors cannot be certain that all posts made during these time periods were retrieved as this may depend on the search function algorithm. A large body of content was, however, retrieved and analysed (an average of 20,721 words for each period).

### Analysis

Longitudinal Facebook group interaction in the form of posts and comments were analysed using psychological discourse analysis to understand how meaning was created in written discourse. This was supplemented by computed-mediated sentiment and linguistic analysis. This approach allowed the combination of thick, qualitative data and big, quantitative data. An examination of the discourse can enhance the capabilities of sentiment analysis (see the discussion section for further details). The analysis focused on what developed and maintained connectedness amongst the users and encouraged them to engage in support and which discursive strategies facilitated the effective communication of public health guidance.

Discourse analysis stemming from discursive theory can be described as an interdisciplinary enterprise with roots in psychology, sociology, and linguistics, and some argue that it lends itself well to naturalistic, rather than researcher-contrived, data (38). The psychological discourse analysis followed the framework proposed by Goodman (39) which involved: deciding on appropriate questions for the analysis, selecting data sources to generate a corpus, searching for action orientation during preliminary reading, generating results through the identification of discursive devices and rhetorical, interactional strategies, and building a case. Goodman suggested that strategies identified within the data may be identified from previous analyses and/or be novel and unique. This required familiarity with the vast discursive literature**.**

The data were collated in the qualitative data analysis software, NVivo 12. A coding system was set up to capture the social actions accomplished within the discourse and associated specific devices, for both administrators and group members. The coding system was developed by pilot coding activities within the research team and drawing on the existing discursive literature. Once consistency in coding was reached, the data were divided into three approximately equal parts and coded by the three researchers. Any ambiguous data or coding queries were discussed and resolved as a team. Each researcher recorded reflective notes throughout the coding process that informed the analysis.

In addition, text files for each period of interest were analysed using the software, Linguistic Inquiry and Word Count, LIWC 2015 (40, 41); LIWC operates by reading text and comparing each word to a dictionary list of words to calculate a percentage of total words in the text that match each dictionary category. The present study focuses on categories relevant to the research questions and exploratory areas of enquiry directed by the discourse analysis (e.g., affect, cognitive, and summary dimension processes) for which no prior assumptions were made. Spearman's rank correlations, conducted in SPSS Statistics 27, were used to examine the variables' relationship with time; this conservative test was appropriate given the small number of observations (i.e., time points).

## Results

**Figure 2:**
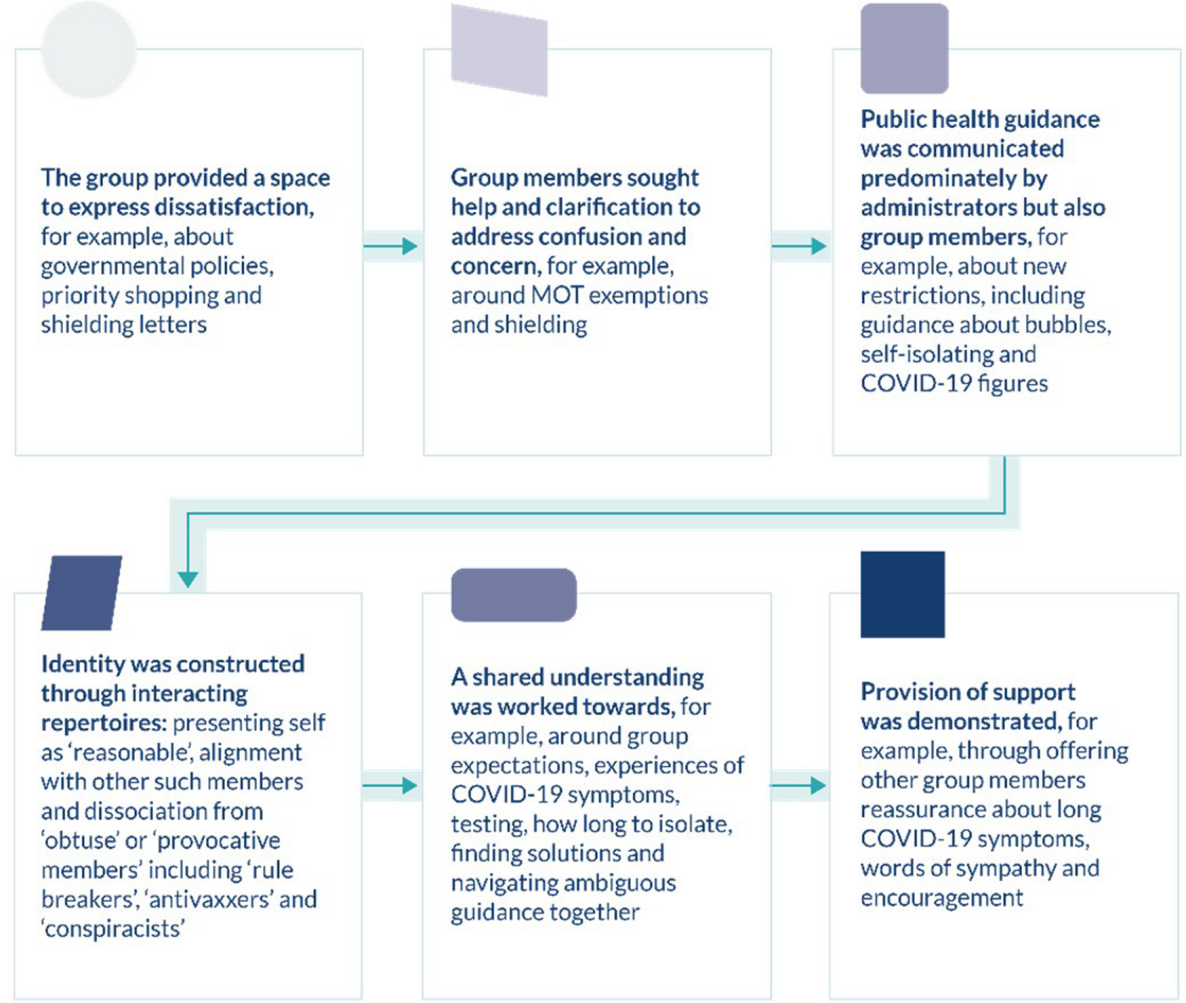
Diagrammatic representation of social actions.

Analyses identified six key social actions that did not operate exclusively at certain time points but rather developed over time (Figure 2). The group provided a space for residents to express their feelings around challenging circumstances and an opportunity to address confusion and concern in times of crisis. The group facilitated the communication of public health messages and promoted health protective behaviours (in line with intervention objectives) through the construction of accepted norms and identities. Interactions worked towards a shared understanding of the COVID-19 evidence base and other group members' perspectives, in turn, mobilising the provision of support in difficult times. These actions were accomplished through the application of specific interactional devices and rhetorical strategies.

In our reporting in the following, specific language analysed at word level is boldfaced and typographical errors within illustrative quotations (i.e., posts authored by members) have been resolved for clarity. For social actions amenable to further computer-mediated investigation, correlations are presented.^2^

### Expression of feeling around challenging circumstances

The group provided a space for residents to express anger and discontent with challenging circumstances. This appeared to be most dominant within the early months of the group’s establishment (March–April 2020), the first lockdown period. However, this recurred at times of changing restrictions, difficulties with priority shopping, and shielding letters. Reflective of this, a negative but non-significant correlation between the time and use of anger emotion words (e.g., hate, annoyed) was found [*r_s_*(5) = –0.67, *p* = .22].

To convey anger and discontent, group members drew on idioms that enabled complex feelings to be expressed with just a few words; the creativity of idioms also stand outs to the reader. In addition to conceptual anger metaphors, endorsement seeking tag-questions (e.g., isn't it), intensifiers (e.g., all), and more “add words” like emphatic swearing and exclamations were used to magnify meaning and amplify the cause for attention. The jocular and cathartic functions of swearing here were used to deal with the perceived problem, whilst arguably building a sense of camaraderie within the group:

it's all a giant pain isn't it (March 2020)

Going on tier 3 in the north they say stay in your local area and no nonessential travel **but in the next breath** they are saying you can move from 3 to 3 in a neighbouring area… **so who bloody knows!** (March 2020)

In addition, disclaimers allowed self-legitimation, portraying the dutiful actions of the writer, and, in doing so, mitigating doubts or pre-empting any negative interpretations of the anger expressed that could detract from the achievement of expressing discontent with societal service provisions:

**This makes my blood boil. All** the money my dad's saved the system by caring for my mum **all** these years and he can’t get any help to do his **bloody** shopping. **Of course [name] and I will do it but** it's not the point. He likes to be independent (March 2020)

### Addressing confusion in times of crisis

Following a discourse dominant in expressed anger, the group appeared to move towards seeking help and clarification to address confusion and concern, for example, around car MOT (the UK required test of car safety and roadworthiness) exemptions and shielding.

This was often achieved through framing posts as queries and the integration of information for the reader, for example, using speaker focused markers (e.g., I know) and complement markers (e.g., so) to signal progression in cognitive processes and elucidate inferences.

In the following example, the group member did not show assumed prior knowledge of the reader, demonstrated by the use of pre-emptive and repetitive synonymous questioning to define a possibly unfamiliar acronym (i.e., SORN).

**Can someone advise please**. MOT Exemption. **I know** it's been mentioned a few times **so thought I’d wait** until after the date to check. My car was due its first MOT yesterday - garage cancelled this last week… Do I temporarily SORN my car? Move it off the road?… (March 2020)

Over time, words used to reference mental state and the hypothesising of others' mental state appeared to be more commonplace, in line with a significant positive correlation found between the time and use of insight words [e.g., think, know; *r_s_*(5) = .90, *p* = .04]:

I don’t disagree, all I would say is, **I imagine** they are so snowed under that **I would think** any challenge, even polite ones, must wear them down. But fair enough if it is out of genuine concern for the good of others. You too have a nice weekend (November 2020)

Maybe **he doesn**’**t want** to because **he's afraid** of giving it to you (November 2020)

### Communicating guidance in a pandemic

Formal public health guidance was predominately communicated by administrators, for example, in June 2020 to relay new restrictions, including guidance about social bubbles, self-isolation, and COVID-19 case numbers. Administrators used several strategies to accomplish this, including the use of conjunctions and temporal adverbs for specificity and transparency around areas of change and uncertainty:

**at the moment** the date for shielding to end is still 30th June. There was an update expected this week. We are **now** no longer expecting that update to happen and it has been **moved to “soon”**. For the time being, you will need to continue holding tight. **As soon as** there is an update, we’ll let you know in here (June 2020)

Factual formulations were employed to present information clearly and signposts to reliable sources of information were provided. This functioned to heighten the credibility of discourse and portray the helpful intentions of administrators:

**Measures announced this week detail** new steps from the government towards easing lockdown restrictions, including the introduction of support bubbles for households with one adult. **The aim of support bubbles is to expand** the support network of those who live alone or are lone parents **to limit** the harmful effects of social isolation. The other measures on 2 m social distancing from others, and only meeting people outside your household **remain**. [**link to government website**]. **FAQs and stock responses**… (June 2020)

Similarly, group members mirrored these strategies:

There you go: to spend time or exercise outdoors—**this should be done locally** wherever possible, but **you can travel** to do so if necessary (for example, **to access** an open space [**government link**]) (November 2020)

As governmental guidance became available, the tendency within group discourse to draw on words for clarity and detail is reflected in the significant positive correlation found between relativity (e.g., area, bend) and time [*r_s_*(5) = 1.00, *p* < .01]. Relativity is categorised as a psychological process and subsumes words related to motion (e.g., arrive, go) and space (e.g., down, in).

### Constructing group identity

Identity was constructed through interacting repertoires that promoted desirable group interaction and protective behaviours. This included framing the self as “reasonable” and dissociation from “obtuse” or “provocative members” including “rule breakers”, “antivaxxers”, and “conspiracists”. This framing, was however, also used by group members as a license to challenge government or authority guidance:

**I am no fan of conspiracy theories or law breaking. However** I think you have to acknowledge that your mandate is to promote a fairly rigid view of the legislation (November 2020)

In turn, administrators employed similar strategies to respond to criticisms, present counter arguments, and dispute the validity of assertions where required, in doing so reinforcing their authority, role obligation, and setting norms and expectations for desirable member characteristics:

**We do have leniency towards those with opposing positions, but** when it becomes misinformation, or unfounded gossip, or just plain “ignore the Law”, we don’t have a choice other than to moderate (November 2020)

The embedding of strong assertions by administrators between greetings (e.g., hello) and down-toners (e.g., stay safe) demonstrated the maintenance of social niceties, set a precedent for appropriate ways to manage disagreement within the group, and prevented possible escalation of conflict. The group member in this example proceeded to communicate agreement and mirrored administrator language, “very glad we agree on this. Stay safe” in doing so, protecting self-image as a “reasonable” member and terminating the exchange. The intensifier (e.g., very) and use of first-person plural pronoun (e.g., we) functioned to strengthen alignment of viewpoint and values.

Similar phrases of agreement, affirmation, thanks, and utterances associated with politeness were employed among group members to promote collegiality and acceptance within the group:

My symptoms were related to my medical condition but they said I had to be tested. It's appalling really as it's so important to get it right. **Glad you were all ok too**.

yes I completely agree x

**Glad you are both ok** and **thanks** for very informative post*.* (June 2020)

In contrast, group members dissociated from third parties with discordant viewpoints, such as employers, using divisive third-person pronouns. This enhanced a sense of group identity by creating “us” vs. “them”:

he should just say to **them** as the government have said people should work from home if **they** are able to (which he is)…

such a joke isnt it. Feel bad for your son…hopefully **they** change **their** minds when more updates come out x

thank you [name]*.* (November 2020)

### Working towards a shared understanding

A key social action that built upon group identity was group members and administrators working together to establish a shared understanding, find solutions, and navigate sometimes ambiguous guidance. This was particularly evident in issues around COVID-19 symptoms, testing, how long to isolate, and childcare.

In order to achieve inter-subjectivity, administrators drew on polite instructive language (e.g., please read) and the notion of expert consensus (e.g., governments across the world) to increase influence. Upon a group member questioning the evidence base for a 14-day virus incubation period, an administrator's use of attitudinal adverbs (e.g., widely, publicly, and even) and adjectives of magnitude (e.g., large, multiple) were also used for rhetoric value and to convey the high degree of certainty towards the proposition, in response to rebuttals:

(Administrator)—[name], this is a **widely** known **publicly** available fact. [two World Health Organization (WHO) links provided]… **please read** the highlighted text, it cites 2 research studies which are linked in the footnotes of the document so that **you can read them in full if you wish**. **Even a basic** Google search will bring up **multiple** other research studies confirming the same. This isn’t a hard to find piece of research to validate and is used by **governments across the world** to inform their self-isolation rules…There are the studies linked to in the WHO article… this study lists **a large number of auxiliary studies** from other countries about incubation period research. Happy reading! (December 2020)

Administrators not only promulgated an understanding of empirical evidence but also aided the interpretation of group member interaction to mitigate and prevent tension arising from potential misunderstandings. For example, when a group member expressed frustration at the use of emojis within an interaction, an administrator wrote,

You may not be aware, but many people with disabilities find using emojis an easier form of communication (November 2020)

The use of the modal auxiliary (e.g., may) to express possibility and the face save (e.g., you may not be aware) functioned to protect the reputation of a group member by politely postulating that such discourse stemmed from a lack of awareness, as opposed to a lack of acceptance of different communication needs and styles.

Administrators also employed face-saving expressions and speech acts such as apologies to supply group members with current information and knowledge and promote shared understanding, in doing so protecting their own professional self-image and appearance of proactivity:

Hello, **I am currently going** through the posts awaiting approval. **Apologies** if there has been a delay in getting to your question. Hope the answer helps [stay safe] (December 2020)

### Providing support in difficult times

Working towards a shared understanding enabled the provision of practical and sometimes emotional support. For example, administrators offered group members reassurance, words of sympathy, and encouragement whilst addressing queries:

(Administrator) … Please see the OP [original post] above with the Government's press release. Hope your Nan recovers well.

Thank you, sadly my Nan has passed

(Administrator)—[name] I am so sorry to read this, (and the delay in getting the answer to you). My sincere condolences to you and your family. (December 2020)

Beyond this, group members demonstrated an increased tendency to draw on authentic personal experiences and narratives, sometimes involving self-disclosure, to offer empathy around issues like long COVID-19 symptoms, personal loss, and financial matters. In accordance, a significant positive correlation was found between time and the dimension, authenticity [*r_s_*(5) = .90, *p* = .04]. Text scoring high in authenticity tends to show people revealing themselves in an honest, spontaneous, and unfiltered way. Use of language conveying access to introspection, thought, and feeling (e.g., feel, hoping) contributed to this:

My sympathies. **We did this** recently—I drove there and my husband drove back. **It was a long, tough day but we (personally)** didn’t feel it necessary to take the risk of staying over when we could manage the trip between us in a day x (November 2020)

In addition, provision of words of encouragement and reassurance drew on positivity and solidarity through shared experiences, often indicated through first-person singular (e.g., I, my) and first-person plural (e.g., we) pronouns. Discourse typifying latter periods of interest at times could be mistaken for conversation among friends who already knew one another:

Hoping it will get better though…

I had covid in March and lost sense of taste and smell for 10 days—**it's back fine.**

My taste came back after around 5 weeks but smell is still hit and miss and I tested positive mid-October. **It's far better than it was though!**

**Came back** in 3 days (January 2021)

It's not always easy to stay positive, everyone will go up and down, **but hopefully when anyone's on a down, we can support each other** and lift back up again (January 2021)

## Discussion

The present study set out to investigate the features of discourse associated with achieving group connectedness and to establish which discursive strategies enabled the communication of public health guidance and promoted desirable interaction. The findings suggest that the group provided a space for Essex residents to express their feelings, in particular anger, around challenging circumstances. This recurred in line with temporal stressors (such as difficulties with shielding and priority shopping) and catalysed opportunities to address confusion and concern within the group during the COVID-19 crisis. The group enabled the dissemination of public health messages and desirable covid protective behaviours through the establishment of accepted social norms and identities. A shared understanding of scientific evidence and members’ perspectives was worked towards mobilising the group to support one another in difficult times.

Findings are in keeping with literature linking the expression of anger with challenges to autonomy (20) and support a relationship between appeals for anger and engagement with online posts (22). Posts expressing anger, sometimes through the use of metaphors and idioms, often elicited empathetic responses in which people shared their problems or experiences to acknowledge confusion or concern. This is not dissimilar to how team members may interact to resolve issues within a work environment (24), albeit more affective in nature. In some cases, self-disclosure gave the appearance of less self-monitoring and social inhibition and promoted authenticity.

In addition, it should be considered that language emotionality can extend beyond the expression of emotion; there was some evidence for development in communicative style and social awareness over time in the group. Development of the use of mental state and insight language is poignant given that infrequent use of linguistic expressions capturing thoughts, emotions, and beliefs has been linked to difficulty with implicit mentalising (42) and poorer wellbeing.

This study exemplifies how within the context of a crisis, individuals can employ technology and personal information to cope with uncertainty. For informational channels to be pursued for this purpose, they need to be perceived as both trusted and credible and require careful regulation (1), in order to ameliorate conspiracy beliefs and promote health-protective behaviours (2, 3).

Administrators achieved this in several ways. The communication of public health messages aimed to be effective, was accomplished through clear and transparent language during times of change in guidance and uncertainty, as advocated by Malecki, Keating and Safdar (7). This also served to uphold professionality within their role and portray genuine intentions, a recognised function of authenticity (31). Discursive devices such as factual formulations and language promoting specificity contributed to this and were mirrored by group members. This is of particular interest given that a greater use of relativity words could be indicative of attention and functioning (43).

Administrators encouraged adherence to issued guidance by presenting accepted and desirable norms within the group, using language linked with politeness and disdain for those endorsing misinformation, or law-breaking activity. The group's use of alignment helped to maintain a positive atmosphere and sense of “togetherness” (27), achieved in part by the use of pronouns to dissociate from criticised third parties and align the self with “reasonable” identities. Early on in the group’s establishment, there was a focus on the expression of sentiment and addressing concerns. However, over time, the construction of identities within the group and the achievement of shared understanding more clearly emerged, allowing a sense of “togetherness” to develop. This movement is in keeping with other work on ECAS; early periods perhaps represent more of an information sharing network than a cohesive community (44).

The importance of reaching a shared understanding to achieve group cohesion has been emphasised (28) and key to this in the present context was administrators tackling incorrect or inappropriate assertions using non-threatening rebuttals. This not only created and protected the comfortable space, but also promoted acceptance within the group, necessary for encouraging help seeking.

This study shows that Facebook can be usefully employed to encourage meaningful interactions during a pandemic and used as a novel approach to engaging the audience as a partner in communication (as opposed to a recipient). The findings support the fact that group interaction should adhere to communication principles based on finding solutions and acknowledging people's concerns (6). A key component of the group’s efficacy was providing Essex residents with the opportunity to access administrators and group members who were willing and able to provide support where required.

This study elucidates specific features, elaborated above, which could be used to guide intervention design and promote audience engagement. For example, actionable strategies for the dissemination of public health messages and support provision might include the use of factual formulations, polite instructions, and language promoting credibility and reputational defence. Group members might draw on insight or mental state language and authenticity to support one another within an online community during times of crisis. In addition to these discursive strategies, additional factors associated with effectiveness and corresponding challenges should be considered, such as speed and agility of dissemination, knowledge of the local context, and administrator personalities (45). The findings could inform the ongoing delivery of the intervention, be applied to a local or regional issue and expanded to other areas of public health beyond COVID-19. Indeed, ECAS has since branched out, creating new groups to address issues such as climate action and the Ukraine crisis.

The suitability of this type of intervention for specific social issues should, however, be examined in future research and approached cautiously. Public health teams should be mindful that engagement and desired intervention characteristics are likely to be influenced by factors such as the degree to which individuals feel stigmatised because of the issue that they are experiencing. Stigma has been associated with several deleterious outcomes rendering support acquisition difficult; there is, however, some evidence that stigma facilitates the use of computer media support, and positive associations have been reported between stigma and the valuing of text-based interaction, anonymous community characteristics, and support from weak ties (10).

While these findings may be useful for guiding future interventions, it should be recognised that they may not be transferable to other social media platforms that, for example, use predominately non-text-based interaction and/or appeal to a different audience in terms of participant demographics. Some discourse markers (e.g., oh, like) are arguably, and perhaps controversially, stylistically stigmatised, for example linked to social status, age, and sex (30). Therefore, the constant evolution of both social media and discourse should be considered in ongoing research activity. Furthermore, given that discursive devices hold cultural and historical significance, further research could usefully examine whether similar findings persist in non-English-speaking online communities. A limitation of this study may be that it is not entirely possible to determine how representative group members were of the overall county of Essex. Although beyond the scope of this study, future research could usefully attempt to stratify participants and investigate different population groups, for example, looking at gender, age, ethnicity, and the extent to which these characteristics influence social action and the linguistic features used.

Nevertheless, a strength of this study was the triangulation of discourse and sentiment findings, which helps to show a holistic picture of multidimensional discursive group functions. The mixed methodology employed militated against restrictions to the predictive accuracy of natural language processing software alone. This is important, as the latter is unlikely to capture the nuances of, for example, metaphor and idiom.

Overall, the ECAS Facebook group enabled social actions to emerge over time through the application of specific interactional devices, indicative of social phenomena such as community cohesion and the development of communication style and social awareness. Public health teams, in designing online community interventions for public health messages and support, should consider employing these interactional devices and may use cited markers to assess developments in group social action. As social media becomes increasingly pivotal in our lives and societal global struggles persist, the use of an online community for public health messaging shows promise for forward thinking, enhancing public engagement and access to support.

## Data Availability

The original contributions presented in the study are included in the article, and further inquiries can be directed to the corresponding author.

## Footnotes

^1^Related study documentation and outputs available: http://phirst.nihr.ac.uk/evaluations/eeabs-evaluation-of-the-impact-of-essex-community-action-site-ecas-upon-attitudes-behaviour-and-public-health-systems-during-the-covid-19-pandemic-essex/ and https://osf.io/va563/?view_only=bde0711960d74d14af9e3400c22986de

^2^Seemingly high values can be attributed to the small number of total observations rendering the occurrence of strong correlations more likely to occur by chance; hence, attention to significance is important in drawing conclusions. It can be problematic to use inferential statistics for these types of data. Alternatives such as assessing differences between means were considered, but Spearman’s correlations most usefully show the relationships between variables and time to supplement the discourse analysis.
